# Supplementary material for: A German Validation of Four Questionnaires Crucial to the Study of Time Perception: BPS, CFC-14, SAQ, and MQT
Source: Int J Environ Res Public Health. 2020 Nov 16;17(22):8477. doi: 10.3390/ijerph17228477 (PMC7696851; doi:10.3390/ijerph17228477)
Supplement: Supplementary file 1 [file ijerph-17-08477-s001.pdf]

VALIDATED GERMAN QUESTIONNAIRES AND ADDITIONAL ANALYSES

SUPPLEMENTARY TABLE 1. *German translation of the original Boredom Proneness Scale (Farmer & Sundberg, 1986) and EFA item allocations to factors.*

Boredom Proneness Scale (BPS)

VPN-Nr.:	Alter:	Geschlecht:	Datum:

Inwieweit sind die folgenden Aussagen für Sie charakteristisch bzw. zutreffend?
Bitte kreuzen Sie für jede Aussage die Zahl an, die Ihrer Antwort entspricht.

ich stimme überhaupt nicht zu	ich stimme teilweise nicht zu		weder noch		ich stimme teilweise zu	ich stimme voll zu
**1**	**2**	**3**	**4**	**5**	**6**	**7**

		**inversed items**	**EFA item allocation to factor**
**01.**	Es fällt mir leicht, mich auf meine Aktivitäten zu konzentrieren.	**inversed**	**external stimulation (F1)**<br>**internal stimulation (F2)**
~~**02.**~~	~~Häufig stelle ich bei der Arbeit fest, dass ich mich um andere Dinge sorge.~~		**deleted**
~~**03.**~~	~~Die Zeit scheint immer langsam zu vergehen.~~		**deleted**
**04.**	Häufig habe ich das Gefühl nicht zu wissen, was ich tun soll.		**internal stimulation (F2)**
**05.**	Ich bin häufig in Situationen, in denen ich sinnlose Dinge tun muss.		**external stimulation (F1)**
~~**06.**~~	~~Es langweilt mich entsetzlich, private Videos oder Urlaubsfotos anderer anzusehen.~~		**deleted**
~~**07.**~~	~~Ich habe immer Aufgaben im Kopf, die ich erledigen muss.~~	**inversed**	**deleted**
**08.**	Ich finde leicht einen Weg mich zu unterhalten.	**inversed**	**internal stimulation (F2)**
**09.**	Viele Dinge, die ich erledigen muss, sind gleichförmig und monoton.		**external stimulation (F1)**
**10.**	Ich brauche mehr Anreiz als Andere, um in Schwung zu kommen.		**external stimulation (F1)**<br>**internal stimulation (F2)**
**11.**	Ich finde Gefallen an den meisten Dingen, die ich tue.	**inversed**	**external stimulation (F1)**<br>**internal stimulation (F2)**
**12.**	Ich bin selten von meiner Arbeit begeistert.		**external stimulation (F1)**
**13.**	In jeder Situation kann ich etwas finden, das mich beschäftigt oder interessiert.	**inversed**	**internal stimulation (F2)**
**14.**	Die meiste Zeit sitze ich herum und tue gar nichts.		**internal stimulation (F2)**
**15.**	Ich kann gut geduldig warten.	**inversed**	**constraint (F3)**

**16.**	Ich weiß häufig nichts mit meiner Zeit anzufangen.		**internal stimulation (F2)**
**17.**	In Situationen, in denen ich warten muss, wie z.B. in einer Schlange stehen, werde ich sehr unruhig.		**constraint (F3)**
~~**18.**~~	~~Ich wache oft mit einer neuen Idee auf.~~	**inversed**	**deleted**
**19.**	Es ist schwer für mich, einen Job zu finden, der aufregend genug ist.		**external stimulation (F1)**
**20.**	Ich hätte gerne mehr aufregende Dinge in meinem Leben.		**external stimulation (F1)**
**21.**	Ich spüre, dass ich die meiste Zeit unter meinen Möglichkeiten arbeite.		**external stimulation (F1)**
~~**22.**~~	~~Viele Menschen würden sagen, dass ich eine kreative und phantasievolle Person bin.~~	**inversed**	**deleted**
**23.**	Ich habe so viele Interessen für die ich alle nicht genug Zeit habe.	**inversed**	**internal stimulation (F2)**
**24.**	Unter meinen Freunden bin ich diejenige/derjenige mit dem größten Durchhaltevermögen.	**inversed**	**internal stimulation (F2)**
**25.**	Außer wenn ich etwas Aufregendes oder gar Gefährliches mache, fühle ich mich leblos und matt.		**internal stimulation (F2)**
**26.**	Es bedarf einer Menge an Veränderung und Abwechslung, um mich wirklich glücklich zu machen.		**external stimulation (F1)**
~~**27.**~~	~~Es scheint so, als ob immer dieselben Dinge im Fernsehen und Filmen zu sehen sind; es wird langweilig.~~		**deleted**
~~**28.**~~	~~Als ich jung war, war ich oft in monotonen und langweiligen Situationen.~~		**deleted**

SUPPLEMENTARY TABLE 2. *German translation of the original Consideration of Future Consequences-14 scale (CFC-14).* ***(F)*** *indicates CFC-future items* ***(I)*** *indicates CFC-immediate items.*

Consideration of Future Consequences-14 (CFC-14)

VPN-Nr.: Alter: Geschlecht: Datum:

Inwieweit sind die folgenden Aussagen für Sie charakteristisch bzw. zutreffend?
Bitte lesen Sie jede Aussage sorgfältig durch
und kreuzen Sie jeweils die Zahl an, die Ihrer Antwort entspricht.

überhaupt nicht charkateristisch für mich	uncharakteristisch für mich		weder noch		charakteristisch für mich	sehr charakteristisch für mich
**1**	**2**	**3**	**4**	**5**	**6**	**7**

**01.**	Ich überlege, wie die Zukunft aussehen könnte und versuche, sie mit meinem täglichen Verhalten zu beeinflussen. **(F)**	**1**	**2**	**3**	**4**	**5**	**6**	**7**
**02.**	Oft verhalte ich mich auf eine bestimmte Art, um Ergebnisse zu erzielen, die vielleicht erst nach Jahren eintreten werden. **(F)**	**1**	**2**	**3**	**4**	**5**	**6**	**7**
**03.**	Ich handle nur um unmittelbare Bedürfnisse zu befriedigen und glaube, dass die Zukunft sich von alleine regeln wird. **(I)**	**1**	**2**	**3**	**4**	**5**	**6**	**7**
**04.**	Mein Verhalten wird nur von unmittelbaren Folgen (d.h. wenige Tage oder Wochen) meiner Handlungen beeinflusst. **(I)**	**1**	**2**	**3**	**4**	**5**	**6**	**7**
**05.**	Meine Bequemlichkeit spielt eine große Rolle für meine Entscheidungen und Handlungen. **(I)**	**1**	**2**	**3**	**4**	**5**	**6**	**7**
**06.**	Ich bin bereit, mein unmittelbares Glück oder Wohlbefinden zu opfern, um zukünftige Ergebnisse zu erzielen. **(F)**	**1**	**2**	**3**	**4**	**5**	**6**	**7**
**07.**	Es ist wichtig, Warnungen vor negativen Folgen ernst zu nehmen, selbst wenn diese erst viele Jahre später eintreffen. **(F)**	**1**	**2**	**3**	**4**	**5**	**6**	**7**
**08.**	Ich glaube, es ist wichtiger, sich nach wichtigen langfristigen Konsequenzen zu richten als nach weniger bedeutsamen, kurzfristigen Folgen zu handeln. **(F)**	**1**	**2**	**3**	**4**	**5**	**6**	**7**
**09.**	Im Allgemeinen ignoriere ich Warnungen über mögliche zukünftige Probleme, weil ich glaube, dass sie gelöst werden, bevor sie kritisch werden. **(I)**	**1**	**2**	**3**	**4**	**5**	**6**	**7**
**10.**	Ich glaube, dass es meist unnötig ist, gegenwärtig auf etwas zu verzichten, da man sich auch später um zukünftige Konsequenzen kümmern kann. **(I)**	**1**	**2**	**3**	**4**	**5**	**6**	**7**
**11.**	Ich handle nur, um unmittelbare Bedürfnisse zu befriedigen und glaube, dass ich mich um eventuell eintretende Probleme später	**1**	**2**	**3**	**4**	**5**	**6**	**7**

	kümmern werde. **(I)**							
**12.**	Da meine tägliche Arbeit konkrete Ergebnisse erbringt, ist diese für mich wichtiger als Verhalten, das langfristige Auswirkungen hat. **(I)**	**1**	**2**	**3**	**4**	**5**	**6**	**7**
**13.**	Wenn ich eine Entscheidung treffe, denke ich darüber nach, wie sie mich in der Zukunft beeinflussen könnte. **(F)**	**1**	**2**	**3**	**4**	**5**	**6**	**7**
**14.**	Mein Verhalten wird im Allgemeinen von zukünftigen Konsequenzen beeinflusst. **(F)**	**1**	**2**	**3**	**4**	**5**	**6**	**7**

SUPPLEMENTARY TABLE 3. *German translation of the Self-Awareness Questionnaire (SAQ).*

**Self-Awareness Questionnaire**			
VPN-Nr.:	Alter:	Geschlecht:	Datum:

Bitte lesen Sie jede Frage aufmerksam durch und wählen Sie die Antwort aus, die am besten beschreibt, wie oft Sie etwas empfinden.

Kreuzen Sie nur eine Antwort an. Es gibt keine richtigen oder falschen Antworten.

**nie**	**manchmal**	**oft**	**sehr oft**	**immer**
**0**	**1**	**2**	**3**	**4**

		nie				immer
**01.**	Ich höre meinen Herzschlag in den Ohren.	**0**	**1**	**2**	**3**	**4**
**02.**	Im Vergleich zu anderen ist mir extrem heiß.	**0**	**1**	**2**	**3**	**4**
**03.**	Ich nehme Schmerz extrem wahr.	**0**	**1**	**2**	**3**	**4**
**04.**	Ich spüre, dass sich mein Magen zusammenzieht.	**0**	**1**	**2**	**3**	**4**
**05.**	Ich verspüre ein plötzliches Hungergefühl.	**0**	**1**	**2**	**3**	**4**
**06.**	Ich habe Rückenschmerzen.	**0**	**1**	**2**	**3**	**4**
**07.**	Ich verspüre ein Kribbeln.	**0**	**1**	**2**	**3**	**4**
**08.**	Ich habe das Gefühl, keine Luft zu bekommen.	**0**	**1**	**2**	**3**	**4**
**09.**	Ich habe Herzklopfen.	**0**	**1**	**2**	**3**	**4**
**10.**	Nach dem Essen fühle ich mich aufgebläht.	**0**	**1**	**2**	**3**	**4**
**11.**	Ich verspüre plötzlich den Drang zu urinieren.	**0**	**1**	**2**	**3**	**4**
**12.**	Ich spüre, dass ich glühe.	**0**	**1**	**2**	**3**	**4**
**13.**	Ich habe Sodbrennen.	**0**	**1**	**2**	**3**	**4**
**14.**	Ich habe Bauchschmerzen.	**0**	**1**	**2**	**3**	**4**
**15.**	Im Vergleich zu anderen ist mir extrem kalt.	**0**	**1**	**2**	**3**	**4**
**16.**	Ich habe das Gefühl, mich übergeben zu müssen.	**0**	**1**	**2**	**3**	**4**
**17.**	Mich überkommt ein Kälteschauer.	**0**	**1**	**2**	**3**	**4**
**18.**	Meine Beine fühlen sich schwer an.	**0**	**1**	**2**	**3**	**4**

		nie				immer
**19.**	Ich habe einen trockenen Hals.	**0**	**1**	**2**	**3**	**4**
**20.**	Meine Brust fühlt sich schwer an.	**0**	**1**	**2**	**3**	**4**
**21.**	Ich habe das Gefühl, dass mir das Herz kurz stehen bleibt.	**0**	**1**	**2**	**3**	**4**
**22.**	Ich verspüre ein plötzliches Durstgefühl.	**0**	**1**	**2**	**3**	**4**
**23.**	Ich atme schwer, ohne mich angestrengt zu haben.	**0**	**1**	**2**	**3**	**4**
**24.**	Meine Ohren glühen.	**0**	**1**	**2**	**3**	**4**
**25.**	Ich habe einen Kloß im Hals.	**0**	**1**	**2**	**3**	**4**
**26.**	Ich habe das Gefühl, ohnmächtig zu werden.	**0**	**1**	**2**	**3**	**4**
**27.**	Ich habe schweißnasse Hände.	**0**	**1**	**2**	**3**	**4**
**28.**	Es fällt mir schwer, zu schlucken.	**0**	**1**	**2**	**3**	**4**

SUPPLEMENTARY TABLE 4. *German translation of the original Metacognitive Questionnaire on Time (MQT) and its original subscales 'others' and 'self' as well as the respective subscale factors 'attention' and 'emotion'.*

**Metacognitive Questionnaire on Time (MQT)**			
VPN-Nr.:	Alter:	Geschlecht:	Datum:

Wir möchten herausfinden, wie **<u>SIE</u>** Zeit wahrnehmen. <u>Es gibt keine falschen oder richtigen Antworten,</u> weil jeder anders ist. Geben Sie möglichst spontan und ehrlich auf **jede** Frage die Antwort, die <u>auf **SIE** zutrifft</u>. Geben Sie jeweils **<u>NUR EINE</u>** Antwort, indem Sie Ihre Auswahl einkreisen.

**stimme gar nicht zu**	**stimme nicht zu**	**weder Zustimmung noch Ablehnung**	**stimme zu**	**stimme völlig zu**
**1**	**2**	**3**	**4**	**5**

**01.**	Wenn sie fröhlich sind, finden die meisten Leute, dass die Zeit schneller vergeht.	others 'emotion'
**02.**	Wenn sie traurig sind, finden die meisten Leute, dass die Zeit schneller vergeht.	others 'emotion' (inversed)
~~**03.**~~	~~In Gegenwart einer fröhlichen Person finden die meisten Leute, dass die Zeit schneller vergeht.~~	~~others 'emotion' (inversed)~~
**04.**	Ich finde, dass die Zeit schneller vergeht, wenn ich etwas Angenehmes tue.	self 'attention'
**05.**	Mir wird die Zeit lang, wenn ich warte.	self 'attention'
**06.**	Es ist mir wichtig, meine Aktivitäten zeitlich organisieren zu können.	self 'attention'
**07.**	Wenn sie traurig sind, haben die meisten Leute das Gefühl, langsamer zu sein.	others 'emotion'
**08.**	Wenn ich irgendwo Schmerzen habe, finde ich, dass die Zeit schneller vergeht.	self 'attention' (inversed)
**09.**	Die meisten Leute finden, dass die Zeit langsamer vergeht, wenn sie sich langweilen.	others 'attention'
~~**10.**~~	~~Die meisten Leute finden, je mehr sie auf die Zeit achten, desto schneller vergeht sie.~~	~~others 'attention' (inversed)~~
**11.**	Den meisten Leuten ist es wichtig, Ihre Aktivitäten zeitlich organisieren zu können.	others 'attention'
**12.**	Die meisten Leute finden, dass die Zeit langsamer vergeht, wenn sie mit Kindern zusammen sind.	others 'attention' (inversed)
**13.**	Den meisten Leuten wird die Zeit lang, wenn sie warten.	others 'attention'

**14.**	Wenn ich fröhlich bin, finde ich, dass die Zeit schneller vergeht.	self ‘emotion‘
**15.**	Je mehr mich begeistert, was ich tue, desto langsamer vergeht die Zeit.	self ‘attention‘ (inversed)
~~**16.**~~	~~Ich finde, dass die Zeit langsamer vergeht, wenn ich mit Kindern zusammen bin.~~	~~self ‘attention‘ (inversed)~~
**17.**	Die meisten Leute finden, dass die Zeit schneller vergeht, wenn sie irgendwo Schmerzen haben.	others ‘attention‘ (inversed)
**18.**	Die meisten Leute finden, je mehr sie begeistert, was sie tun, desto langsamer vergeht die Zeit.	others ‘attention‘ (inversed)
~~**19.**~~	~~In Gegenwart einer fröhlichen Person finde ich, dass die Zeit schneller vergeht.~~	~~self ‘emotion‘ (inversed)~~
**20.**	Ich finde, dass die Zeit langsamer vergeht, wenn ich mich langweile.	self ‘attention‘
**21.**	Wenn ich traurig bin, habe ich das Gefühl, langsamer zu sein.	self ‘emotion‘
**22.**	Wenn ich traurig bin, finde ich, dass die Zeit schneller vergeht.	self ‘emotion‘ (inversed)
**23.**	Die meisten Leute finden, dass die Zeit schneller vergeht, wenn sie etwas Angenehmes tun.	others ‘attention‘
**24.**	Ich finde, je mehr ich auf die Zeit achte, desto schneller vergeht sie.	self ‘attention‘ (inversed)

SUPPLEMENTARY TABLE 5. *EFA two factorial solution: communalities and factor loadings after the direct oblimin rotation of the shortened CFC-10.*

Item no.	communality (h^2)	factor 1	factor 2
01 (CFC-F)	.60	**-.76**	-.21
02 (CFC-F)	.53	**-.73**	-.29
03 (CFC-I)	.52	**.68**	**.53**
04 (CFC-I)	.32	**.54**	**.40**
07 (CFC-F)	.27	-.30	**-.52**
09 (CFC-I)	.52	.23	**.71**
10 (CFC-I)	.41	.26	**.64**
11 (CFC-I)	.54	**.49**	**.72**
13 (CFC-F)	.25	**-.42**	**-.43**
14 (CFC-F)	.34	**-.53**	**-.45**
**eigenvalue**		**3.91**	**1.43**
**variance**	**53.44**	**39.12**	**14.33**

SUPPLEMENTARY TABLE 6. *EFA communalities and factor loadings after the direct oblimin rotation of the SAQ items.*

Item no.	communality (h^2)	factor 1	factor 2
04	.28	**.53**	.31
06	.16	**.40**	.19
07	.26	**.49**	.13
08	.48	**.69**	.27
09	.37	**.61**	.30
10	.22	**.40**	**.40**
14	.33	**.48**	**.50**
15	.45	**.64**	**.67**
16	.31	**.48**	**.47**
17	.37	**.48**	**.56**
18	.34	**.57**	.38
20	.39	**.62**	.28
21	.32	**.56**	.19
23	.18	**.43**	.20
25	.27	**.51**	.31
26	.34	**.52**	**.48**
28	.24	**.47**	.35
**eigenvalue**		**5.28**	**1.31**
**% of variance**	**38.80**	**31.08**	**7.72**
**cronbach's alpha**	**.84**	.84	**.70**

Note. Factor loadings < .40 are suppressed in this table.

SUPPLEMENTARY TABLE 7. *EFA two factorial solution after the direct oblimin rotation of the MQT 'others' items after having removed items with low communality and factor loadings.*

Item no.	communality (h^2)	factor 1	factor 2
01	.46	.36	**-.67**
12*	.27	**.51**	-.14
17*	.24	**.47**	-.32
18*	.46	**.67**	-.35
23	.53	.27	**-.73**
**eigenvalue**		**2.04**	**1.09**
**variance**	**62.49**	**40.74**	**21.75**
**cronbach's alpha**	**.62**	**.56**	**.65**

Note. * items have been inversed before conducting the EFA.

SUPPLEMENTARY TABLE 8. *EFA two factorial solution after the direct oblimin rotation of the MQT 'self' items after having removed items with low communality and factor loadings.*

Item no.	communality (h^2)	factor 1	factor 2
04	.34	**.53**	**.42**
05	.37	.14	**.60**
14	.70	**.83**	.25
15*	.19	**.43**	.13
20	.39	**.42**	**.58**
**eigenvalue**		**2.05**	**1.08**
**variance**	**62.50**	**40.96**	**21.54**
**cronbach's alpha**	**.61**	**.63**	**.54**

Note. * items have been inversed before conducting the EFA.

SUPPLEMENTARY TABLE 9. *Final German version of the Boredom Proneness Scale (BPS-18).*

**Boredom Proneness Scale (BPS-18)**			
VPN-Nr.:	Alter:	Geschlecht:	Datum:

Inwieweit sind die folgenden Aussagen für Sie charakteristisch bzw. zutreffend?
Bitte kreuzen Sie für jede Aussage die Zahl an, die Ihrer Antwort entspricht.

ich stimme überhaupt nicht zu	ich stimme teilweise nicht zu		weder noch		ich stimme teilweise zu	ich stimme voll zu
**1**	**2**	**3**	**4**	**5**	**6**	**7**

		stimme überhaupt nicht zu						stimme voll zu
**01.**	Es fällt mir leicht, mich auf meine Aktivitäten zu konzentrieren.	**1**	**2**	**3**	**4**	**5**	**6**	**7**
**02.**	Häufig habe ich das Gefühl nicht zu wissen, was ich tun soll.	**1**	**2**	**3**	**4**	**5**	**6**	**7**
**03.**	Ich bin häufig in Situationen, in denen ich sinnlose Dinge tun muss.	**1**	**2**	**3**	**4**	**5**	**6**	**7**
**04.**	Ich finde leicht einen Weg mich zu unterhalten.	**1**	**2**	**3**	**4**	**5**	**6**	**7**
**05.**	Viele Dinge, die ich erledigen muss, sind gleichförmig und monoton.	**1**	**2**	**3**	**4**	**5**	**6**	**7**
**06.**	Ich brauche mehr Anreiz als Andere, um in Schwung zu kommen.	**1**	**2**	**3**	**4**	**5**	**6**	**7**
**07.**	Ich finde Gefallen an den meisten Dingen, die ich tue.	**1**	**2**	**3**	**4**	**5**	**6**	**7**
**08.**	Ich bin selten von meiner Arbeit begeistert.	**1**	**2**	**3**	**4**	**5**	**6**	**7**
**09.**	In jeder Situation kann ich etwas finden, das mich beschäftigt oder interessiert.	**1**	**2**	**3**	**4**	**5**	**6**	**7**
**10.**	Die meiste Zeit sitze ich herum und tue gar nichts.	**1**	**2**	**3**	**4**	**5**	**6**	**7**
**11.**	Ich kann gut geduldig warten.	**1**	**2**	**3**	**4**	**5**	**6**	**7**
**12.**	Ich weiß häufig nichts mit meiner Zeit anzufangen.	**1**	**2**	**3**	**4**	**5**	**6**	**7**
**13.**	In Situationen, in denen ich warten muss, wie z.B. in einer Schlange stehen, werde ich sehr unruhig.	**1**	**2**	**3**	**4**	**5**	**6**	**7**
**14.**	Es ist schwer für mich, einen Job zu finden, der aufregend genug ist.	**1**	**2**	**3**	**4**	**5**	**6**	**7**
**15.**	Ich hätte gerne mehr aufregende Dinge in meinem Leben.	**1**	**2**	**3**	**4**	**5**	**6**	**7**
**16.**	Ich spüre, dass ich die meiste Zeit unter meinen Möglichkeiten arbeite.	**1**	**2**	**3**	**4**	**5**	**6**	**7**
**17.**	Ich habe so viele Interessen für die ich alle nicht genug Zeit habe.	**1**	**2**	**3**	**4**	**5**	**6**	**7**
**18.**	Unter meinen Freunden bin ich diejenige/derjenige mit dem größten Durchhaltevermögen.	**1**	**2**	**3**	**4**	**5**	**6**	**7**

**19.**	Außer wenn ich etwas Aufregendes oder gar Gefährliches mache, fühle ich mich leblos und matt.	**1**	**2**	**3**	**4**	**5**	**6**	**7**
**20.**	Es bedarf einer Menge an Veränderung und Abwechslung, um mich wirklich glücklich zu machen.	**1**	**2**	**3**	**4**	**5**	**6**	**7**

SUPPLEMENTARY TABLE 10. *Final German version of the Consideration of Future Consequences scale (CFC-10).*

Consideration of Future Consequences (CFC-10)

VPN-Nr.: Alter: Geschlecht: Datum:

Inwieweit sind die folgenden Aussagen für Sie charakteristisch bzw. zutreffend?
Bitte lesen Sie jede Aussage sorgfältig durch
und kreuzen Sie jeweils die Zahl an, die Ihrer Antwort entspricht.

überhaupt nicht charkateristisch für mich	uncharakteristisch für mich		weder noch		charakteristisch für mich	sehr charakteristisch für mich
**1**	**2**	**3**	**4**	**5**	**6**	**7**

**01.**	Ich überlege, wie die Zukunft aussehen könnte und versuche, sie mit meinem täglichen Verhalten zu beeinflussen.	**1**	**2**	**3**	**4**	**5**	**6**	**7**
**02.**	Oft verhalte ich mich auf eine bestimmte Art, um Ergebnisse zu erzielen, die vielleicht erst nach Jahren eintreten werden.	**1**	**2**	**3**	**4**	**5**	**6**	**7**
**03.**	Ich handle nur um unmittelbare Bedürfnisse zu befriedigen und glaube, dass die Zukunft sich von alleine regeln wird.	**1**	**2**	**3**	**4**	**5**	**6**	**7**
**04.**	Mein Verhalten wird nur von unmittelbaren Folgen (d.h. wenige Tage oder Wochen) meiner Handlungen beeinflusst.	**1**	**2**	**3**	**4**	**5**	**6**	**7**
**05.**	Es ist wichtig, Warnungen vor negativen Folgen ernst zu nehmen, selbst wenn diese erst viele Jahre später eintreffen.	**1**	**2**	**3**	**4**	**5**	**6**	**7**
**06.**	Im Allgemeinen ignoriere ich Warnungen über mögliche zukünftige Probleme, weil ich glaube, dass sie gelöst werden, bevor sie kritisch werden.	**1**	**2**	**3**	**4**	**5**	**6**	**7**
**07.**	Ich glaube, dass es meist unnötig ist, gegenwärtig auf etwas zu verzichten, da man sich auch später um zukünftige Konsequenzen kümmern kann.	**1**	**2**	**3**	**4**	**5**	**6**	**7**
**08.**	Ich handle nur, um unmittelbare Bedürfnisse zu befriedigen und glaube, dass ich mich um eventuell eintretende Probleme später kümmern werde.	**1**	**2**	**3**	**4**	**5**	**6**	**7**
**09.**	Wenn ich eine Entscheidung treffe, denke ich darüber nach, wie sie mich in der Zukunft beeinflussen könnte.	**1**	**2**	**3**	**4**	**5**	**6**	**7**
**10.**	Mein Verhalten wird im Allgemeinen von zukünftigen Konsequenzen beeinflusst.	**1**	**2**	**3**	**4**	**5**	**6**	**7**

SUPPLEMENTARY TABLE 11. *Final German version of the Self-Awareness Questionnaire (SAQ-17).*

Self-Awareness Questionnaire

VPN-Nr.: Alter: Geschlecht: Datum:

Bitte lesen Sie jede Frage aufmerksam durch und wählen Sie die Antwort aus, die am besten beschreibt, wie oft Sie etwas empfinden.

Kreuzen Sie nur eine Antwort an. Es gibt keine richtigen oder falschen Antworten.

nie	manchmal	oft	sehr oft	immer
0	1	2	3	4

		nie				immer
**01.**	Ich höre meinen Herzschlag in den Ohren.	**0**	**1**	**2**	**3**	**4**
**02.**	Ich spüre, dass sich mein Magen zusammenzieht.	**0**	**1**	**2**	**3**	**4**
**03.**	Ich verspüre ein Kribbeln.	**0**	**1**	**2**	**3**	**4**
**04.**	Ich habe das Gefühl, keine Luft zu bekommen.	**0**	**1**	**2**	**3**	**4**
**05.**	Ich habe Herzklopfen.	**0**	**1**	**2**	**3**	**4**
**06.**	Nach dem Essen fühle ich mich aufgebläht.	**0**	**1**	**2**	**3**	**4**
**07.**	Ich spüre, dass ich glühe.	**0**	**1**	**2**	**3**	**4**
**08.**	Ich habe Bauchschmerzen.	**0**	**1**	**2**	**3**	**4**
**09.**	Ich habe das Gefühl, mich übergeben zu müssen.	**0**	**1**	**2**	**3**	**4**
**10.**	Mich überkommt ein Kälteschauer.	**0**	**1**	**2**	**3**	**4**
**11.**	Meine Beine fühlen sich schwer an.	**0**	**1**	**2**	**3**	**4**
**12.**	Meine Brust fühlt sich schwer an.	**0**	**1**	**2**	**3**	**4**
**13.**	Ich habe das Gefühl, dass mir das Herz kurz stehen bleibt.	**0**	**1**	**2**	**3**	**4**
**14.**	Ich atme schwer, ohne mich angestrengt zu haben.	**0**	**1**	**2**	**3**	**4**
**15.**	Ich habe einen Kloß im Hals.	**0**	**1**	**2**	**3**	**4**
**16.**	Ich habe das Gefühl, ohnmächtig zu werden.	**0**	**1**	**2**	**3**	**4**
**17.**	Es fällt mir schwer, zu schlucken.	**0**	**1**	**2**	**3**	**4**

SUPPLEMENTARY TABLE 12. *Final German version of the Metacognitive Questionnaire on Time (MQT-20).*

**Metacognitive Questionnaire on Time (MQT)**			
VPN-Nr.:	Alter:	Geschlecht:	Datum:

Wir möchten herausfinden, wie **SIE** Zeit wahrnehmen. Es gibt keine falschen oder richtigen Antworten, weil jeder anders ist. Geben Sie möglichst spontan und ehrlich auf **jede** Frage die Antwort, die auf **SIE** zutrifft. Geben Sie jeweils **NUR EINE** Antwort, indem Sie Ihre Auswahl einkreisen.

**stimme gar nicht zu**	**stimme nicht zu**	**weder Zustimmung noch Ablehnung**	**stimme zu**	**stimme völlig zu**
**1**	**2**	**3**	**4**	**5**

		stimme gar nicht zu				stimme völlig zu
**01.**	Wenn sie fröhlich sind, finden die meisten Leute, dass die Zeit schneller vergeht.	**1**	**2**	**3**	**4**	**5**
**02.**	Wenn sie traurig sind, finden die meisten Leute, dass die Zeit schneller vergeht.	**1**	**2**	**3**	**4**	**5**
**03.**	Ich finde, dass die Zeit schneller vergeht, wenn ich etwas Angenehmes tue.	**1**	**2**	**3**	**4**	**5**
**04.**	Mir wird die Zeit lang, wenn ich warte.	**1**	**2**	**3**	**4**	**5**
**05.**	Es ist mir wichtig, meine Aktivitäten zeitlich organisieren zu können.	**1**	**2**	**3**	**4**	**5**
**06.**	Wenn sie traurig sind, haben die meisten Leute das Gefühl, langsamer zu sein.	**1**	**2**	**3**	**4**	**5**
**07.**	Wenn ich irgendwo Schmerzen habe, finde ich, dass die Zeit schneller vergeht.	**1**	**2**	**3**	**4**	**5**
**08.**	Die meisten Leute finden, dass die Zeit langsamer vergeht, wenn sie sich langweilen.	**1**	**2**	**3**	**4**	**5**
**09.**	Den meisten Leuten ist es wichtig, Ihre Aktivitäten zeitlich organisieren zu können.	**1**	**2**	**3**	**4**	**5**
**10.**	Die meisten Leute finden, dass die Zeit langsamer vergeht, wenn sie mit Kindern zusammen sind.	**1**	**2**	**3**	**4**	**5**
**11.**	Den meisten Leuten wird die Zeit lang, wenn sie warten.	**1**	**2**	**3**	**4**	**5**
**12.**	Wenn ich fröhlich bin, finde ich, dass die Zeit schneller vergeht.	**1**	**2**	**3**	**4**	**5**
**13.**	Je mehr mich begeistert, was ich tue, desto langsamer vergeht die Zeit.	**1**	**2**	**3**	**4**	**5**

		stimme gar nicht zu				stimme völlig zu
**14.**	Die meisten Leute finden, dass die Zeit schneller vergeht, wenn sie irgendwo Schmerzen haben.	**1**	**2**	**3**	**4**	**5**
**15.**	Die meisten Leute finden, je mehr sie begeistert, was sie tun, desto langsamer vergeht die Zeit.	**1**	**2**	**3**	**4**	**5**
**16.**	Ich finde, dass die Zeit langsamer vergeht, wenn ich mich langweile.	**1**	**2**	**3**	**4**	**5**
**17.**	Wenn ich traurig bin, habe ich das Gefühl, langsamer zu sein.	**1**	**2**	**3**	**4**	**5**
**18.**	Wenn ich traurig bin, finde ich, dass die Zeit schneller vergeht.	**1**	**2**	**3**	**4**	**5**
**19.**	Die meisten Leute finden, dass die Zeit schneller vergeht, wenn sie etwas Angenehmes tun.	**1**	**2**	**3**	**4**	**5**
**20.**	Ich finde, je mehr ich auf die Zeit achte, desto schneller vergeht sie.	**1**	**2**	**3**	**4**	**5**
